# Experiment and Simulation of a Selective Subwavelength Filter with a Low Index Contrast

**DOI:** 10.3390/nano9101497

**Published:** 2019-10-21

**Authors:** Tao Li, Mohamed Asbahii, Jian-Yee Lim, Hong Xie, Chan-Wai Koh, Min-Hao Goh, Kian-Soo Ong, Hang Zhang, Ding Ding

**Affiliations:** 1 Institute of Engineering Thermophysics, Chinese Academy of Sciences, University of Chinese Academy of Sciences, Beijing 100049, China; litao@iet.cn; 2 University of Chinese Academy of Sciences, Beijing 100049, China; 3 Institute of Material Research and Engineering, A*STAR Research Entities, 1 Fusionopolis Way, Singapore 138632, Singapore; asbahim@imre.a-star.edu.sg (M.A.); ks-ong@imre.a-star.edu.sg (K.-S.O.); 4 Singapore Institute of Manufacturing Technology, A*STAR Research Entities, 1 Fusionopolis Way, Singapore 138632, Singapore; jianyee1998@gmail.com (J.-Y.L.); hxie@simtech.a-star.edu.sg (H.X.); kohcw@simtech.a-star.edu.sg (C.-W.K.); gohmh@simtech.a-star.edu.sg (M.-H.G.)

**Keywords:** contrast, nanophotonics, grating, selective

## Abstract

Subwavelength gratings have been of great interest recently due to their ability to eliminate multiple orders. However, high index contrast (Δn∼3) is typically achieved using metals or high-index dielectrics surrounded by vacuum in order to maintain good optical selectivity. Here, we theoretically propose and experimentally realize a selective subwavelength grating using an index contrast of Δn∼1.2 without vacuum. Despite its low index contrast, our simulation and experiments show that good optical selectivity is achieved using the same physics as subwavelength gratings made of high-index contrast. Such polymer-based encapsulated gratings are easier to scale up for use in large-area applications such as photovoltaics and lighting.

## 1. Introduction

Angular and wavelength selectivity of light has enabled many applications [1,2], ranging from managing thermal radiation [3] to directing solar irradiation on windows [4]. Advances in nanofabrication have led to the realization of microstructures that mimics traditional diffraction gratings but on the scale of the wavelength of light or smaller [5]. Such gratings have led to the developments of selective optical elements [6,7,8,9,10,11,12] due to the elimination of non-zero order diffraction modes with increased coupling efficiency. Subwavelength gratings have been successfully applied to achieve angular selectivity both in the near infrared [13] and mid-infrared domains [14,15] while metallic sub-wavelength gratings have been used to realize polarizers and phase shifters in a wide range of frequency domains [16,17,18,19,20]. Due to losses in metals, recent interest has been focused on developing dielectric sub-wavelength gratings with high dielectric constant surrounded by vacuum [21] commonly known as high contrast gratings (HCG). Here we define the difference between the highest index material and the lowest index in the grating as Δn. For instance, a silicon subwavelength grating will have an index contrast of around Δn∼3 in the visible regime. The high index contrast is important to allow for good performance of HCGs [22]. There have been designs [23,24] in which a thin layer of substrate is the same material as the grating that is used to support the HCG. It is however still difficult to make HCGs which are suspended in vacuum for large-scale applications compared to polymer-based materials.

Nanoimprinting has enabled the manufacturing of controllable nano-patterns on a large scale [25]. In fact, the concept of nanoimprinting large scale photonic structures has already been proposed for photon managements in solar cells [26]. The concept of photon management is widely discussed in spontaneous downconversion processes for augmented photosynthesis [27] and enhancing LED efficiency [19,28]. These applications typically require both angular and wavelength selectivity. Large scale realizations of angular selectivity have so far been primarily based on quasi-random sub-wavelength structures [4]. Nanostructures embedded in a polymer layer are compatible with nanoimprinting, making it easier to fabricate on a large scale. However, the index contrast available for these polymer-based gratings is reduced compared to HCGs. Here, we theoretically propose and experimentally demonstrate the use of encapsulated polymer-glass design to create a sub-wavelength grating structure with good optical selectivity. Due to the lower-index contrast in our design compared to HCGs, we use the term low-contrast gratings (LCG) to describe our sub-wavelength gratings. Contrary to the assumption that high contrast is necessary for good optical selectivity, our gratings is made from glass, polymers and titanium dioxide, giving an index contrast of Δn∼1.2 in the visible regime [29,30]. Meanwhile, we also utilize methods such as modal analysis [31] and effective medium theory (EMT) [32,33], typically used for HCGs, to predict optical properties of LCGs. We found that LCGs are well-described by both methods, provide good optical selectivity, and are much less sensitive to changes in structural parameters such as grating height compared to HCGs.

## 2. Materials and Methods

In this work, we aim to develop a transparent grating that reflects the visible wavelength around 530 nm at zero degrees of incidence but is transparent at other angles and wavelengths. Such a structure is potentially useful for light trapping of directly incident light for applications such as augmented photosynthesis [27], white light generation with single color light emitting diodes [28] and spectral conversion for single band solar cells [34]. We simulate our design using a commercial finite-difference time-domain (FDTD) simulator [35]. Optimization for dimensions of the structure was carried using particle swarm method [36] to minimize transmission at zero degrees over the wavelength range around 520 nm. The incident light is assumed to be polarized perpendicular to the grating. Next, we experimentally fabricated LCG samples using the same material choice and comparable aspect ratios to the optimized structure. The samples are fabricated by depositing Titanium Dioxide using Chemical Vapour Deposition on a glass substrate. To prevent charging effects in electron beam lithography, a thin layer of Chromium is deposited in between glass and Titanium Dioxide. The patterned area is typically a few millimeters in length and width. Then, we deposit a layer of photoresist followed by electron beam lithography, etching and lift-off. The prepared samples are characterized with Dimension Icon Atomic Force Microscope (AFM) from Bruker before a layer of Poly(methyl methacrylate) (PMMA) is deposited on it. The optical transmission of the sample was measured using a Perkin Elmer UV-Visible Spectrometer with an angle adjustable attachment. The polarization dependent zero-angle transmission was measured using a spectroscopic ellipsometer (V-VASE from J. A. Woollam Co.). The incident beam is linearly polarized and the beam size is set around 2 mm in diameter for the measurement. To understand the origin of these highly reflective modes, we use the modal analysis [31,37] so understand the number of modes existing in our system. We also use the EMT in Ref. [33] to characterize our LCG. Both methods cannot accurately simulate the exact optical properties but serve as good guidance for designing LCGs.

## 3. Results

**Figure 1:**
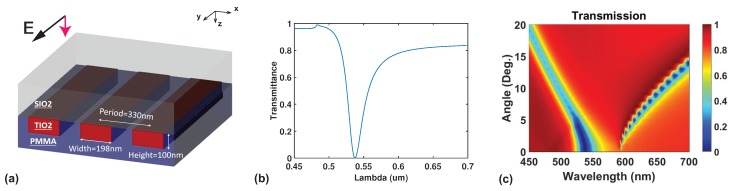
(**a**) Schematic of our proposed angular selective periodic grating structure optimized for selective reflection. The top encapsulating layer is silicon dioxide and the titanium dioxide grating structure is embedded in Poly(methyl methacrylate) (PMMA). Each titanium dioxide unit is 198 nm wide and 100 nm tall with a fill fraction of 0.6. The polarization of the incident electric field (black arrow labeled TE) is along the y direction and the field is propagation in the z direction (red arrow). (**b**) Transmission versus wavelength at normal incidence. It can be seen that there exist a highly reflective region around the wavelength range of 540 nm. (**c**) Plot of transmission versus wavelength at all angles of incidence. It can be seen that transmission at all angles is limited to two narrow bands, one occurring from 450–500 nm and another from 600 nm onwards.

Figure 1a shows an illustration of our optimized design with the condition of the incident electric field being polarized in the y direction. We choose glass as the top material and PMMA as the bottom material to provide a fully encapsulated design which is robust for ambient applications. The dimensions of the titanium dioxide lines are optimized for minimal transmission at zero degrees over the wavelength range around 540 nm with a fill fraction of 0.6. Figure 1b shows the simulated transmission versus wavelength plot at zero angle of incidence. The designed sub-wavelength structure was capable of achieving above 80% transmission at all wavelengths except for a highly reflective region around the wavelength range of 540 nm. If we look at the transmission at other angles of incidence in Figure 1c, we observe a fairly consistent reflection window at 540 nm from zero to five degrees before the window narrows and becomes wavelength dependent. As such, this filter is capable of blocking out directly incident light at 540 nm but transmitting off-angle scattered light at the same wavelength. At the same time, Figure 1c shows a narrow reflection window which emerges beyond 600 nm at higher angles of incidence. Overall, the designed LCG is transparent over much of the wavelength range and angles of incidence. This makes LCGs suitable for designing filters where only one angle-selective reflection window is desired over a board range of wavelengths.

**Figure 2:**
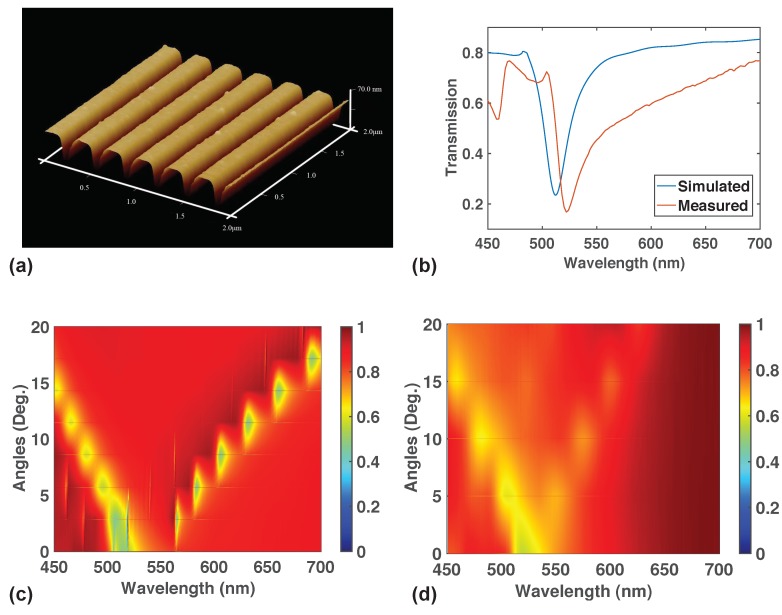
(**a**) Atomic Force Microscope (AFM) image of a fabricated sample. The height of the lines are measured to the around 60 nm while the period of the samples have been measured to be around 320 nm. (**b**) Comparison of p-polarized transmission versus wavelength at zero-angle from experiment and simulation. (**c**) Unpolarized simulation of transmission of a sample with the same dimensions as (**a**). (**d**) Measured transmission as a function of angle and wavelength with unpolarized source. It can be seen that (**c**,**d**) are in relatively good agreement with each other.

We went further to experimentally fabricate the proposed grating. Figure 2a shows the AFM images of one of the fabricated gratings without the PMMA top layer. The depth of the grating was measured to be around 50 nm with a pitch of 320 nm, with a measured fill fraction of around 0.7. The entire sample can be as big as 5 mm by 5 mm. This is close to the design in Figure 1 but there are differences between them. First, the pitch is 50 nm instead of 100 nm due to the challenges in fabrication involved in producing successful lift-off when the height is increased. Second, there is a thin layer of Chromium (between 1–5 nm) in between glass and titanium dioxide due to the need to have a conducting substrate for electron-beam lithography.

Using the dimensions of the fabricated sample from AFM measurement inclusive of the Chromium layer, we simulated the optical transmission as a function of angle in FDTD for both polarizations. Figure 2b shows the p-polarized optical transmission from simulation and experiment at normal incidence. It can be seen that there is some good agreement between them especially in position and the shape of the transmission dip around 520 nm. The peak transmission values are slightly different between experiment and simulation. This is due to the fact that the simulation assumed a semi-infinite PMMA layer while the experimental measurement was performed with PMMA of a finite thickness of a few hundred micrometers. Simulating such a thick layer of PMMA was not computationally feasible with our available computing resources. We would like to emphasize that we have yet to optimize the fabrication steps and parameters and we are already able to obtain reasonably some good agreement between theory and experiment. Furthermore, we have yet to examine the effects of the shape of the sidewalls on the transmission profiles. The measurement was also performed using ellipsometer and UV-Visible spectrometers with beam sizes comparable to the patterned area. Thus, there is good homogeneity of the fabricated gratings throughout the sample over a large area. We believe that this result indicates to a promising outlook for fabricating such structures with nanoimprinting.

**Figure 3:**
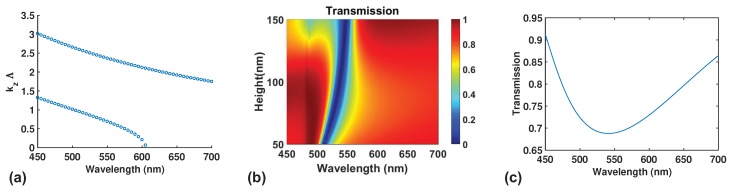
(**a**) Modal propagation vector in the z direction as a function of wavelength of the propagating modes in the grating structure. There is a cutoff wavelength near 600 nm below which a second mode begin to exist below this wavelength. (**b**) Plot of transmission versus wavelength for different heights of the grating structure. A reflective region exists over a wide range of height of gratings around the wavelength range of 500–550 nm. (**c**) Effective medium theory (EMT) calculation of the grating structure in Figure 1a. The minimum transmission corresponds well with the minimum position in (**b**) at a height of 100 nm.

**Figure 4:**
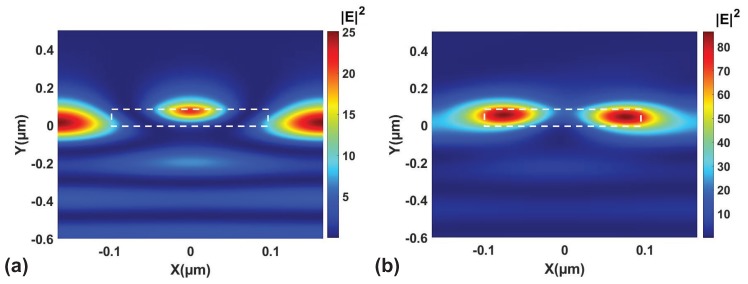
Cross section electric field profile at the reflection windows in Figure 1c at (**a**) 0 degrees at 538 nm and (**b**) 5 degrees at 613nm, respectively. The high index titanium dioxide region is labelled with a white dashed box. There are two branches dictated by the cutoff in Figure 3a. The field profile in (**a**) is localized outside the high index region while they are below the cutoff wavelength, representing the dual mode regime in the dispersion in Figure 3a. The field profile in (**b**) is localized inside the high index region as they are above the cutoff wavelength, representing the single mode regime in the dispersion in Figure 3a.

The simulated transmission after averaging the polarization is given in Figure 2c. Then, we performed angle-dependent transmission measurements using unpolarized source and the results are plotted in Figure 2d. We can see that some good agreement has been obtained between the two. The branching of two transmission dips in experimental measurements also shows the presence of two modes discussed in Figure 3 and Figure 4. There is also some disagreement between the angle dependence of the second mode at higher wavelength between theory and experiment. This could be due to slight geometrical differences between the simulated and the actual geometry.

## 4. Discussion

To understand more about the nature of the selective reflectance, we use modal analysis to obtain the resulting dispersion in the z direction $k_{z}$ multiplied by the period Λ versus wavelength. This is shown in Figure 3a for the same structure show in Figure 1. A second mode starts to emerge below the cutoff wavelength of 600 nm, causing the LCG to be operating in the dual mode regime. In this regime, dual mode interference lead to broadband reflectance at normal incidence as explained and observed in various references [31,37,38]. However, the region of high reflectance in HCGs under dual mode interference varies with the height of the grating in a very complicated fashion where a slight variation in thickness of the gratings makes a great difference in where the reflection windows are positioned [31,37]. This is not the case in our LCG as shown in Figure 3b. The region of high reflection is almost independent of the wavelength for different grating heights up to 1.5 times the period of the optimized design. Thus, the dual mode interference in LCGs is not as sensitive to the change in grating height compared to typical HCGs.

To recap, in Figure 3a there exist a cutoff wavelength below which there are two modes and above which there is only one mode. In Figure 1, we have two windows of reflectance, one below the cutoff wavelength and one above the cutoff wavelength. To understand the distinction between the two regimes, we examine the electric field profiles at these reflectance maxima. The field profile at maximum reflection at zero and five degrees of incidence and its electric field intensity profile is shown in Figure 4a,b, respectively. It can be seen that the field profile is one where the field intensity is outside the high index material with a small region in the middle to top region of the high index material. This is somewhat in agreement with field profile of HCGs in the dual mode regime [37]. Thus, it is possible to have angular and wavelength selectivity in the dual mode regime as well as in the single mode regime as described by Refs. [31,37].

**Figure 5:**
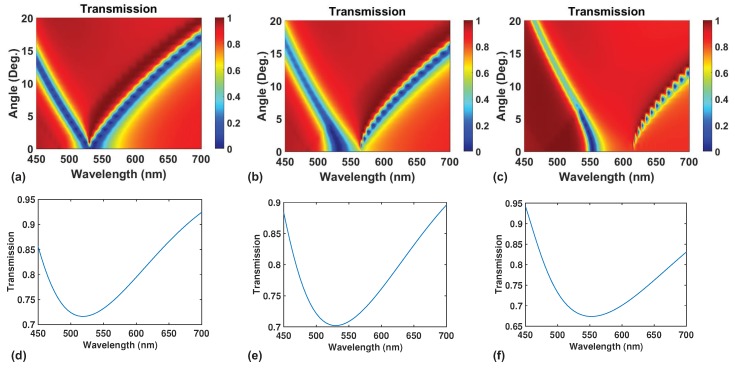
Transmission versus angle plots for different fill fractions of (**a**) 0.4, (**b**) 0.5 and (**c**) 0.7. The corresponding transmission versus wavelength at zero incidence are calculated using effective medium theory (EMT) for the same set of fill fractions of (**d**) 0.4, (**e**) 0.5 and (**f**) 0.7. EMT is able to describe the positions of maximum transmission at zero angle as shown in (**a**–**c**).

If we now vary the fill fraction of the grating from 0.4 to 0.7 while keeping the period fixed at 330 nm, we observe that the position of the reflection window changes accordingly as shown in Figure 5a–c. The position of the maximum reflection changes with wavelength and the width of the high reflection region also reduces with increasing filling fraction. One can use fill fraction to tune the position and the width of the reflection window. Mode analysis like the one in Figure 3a reflect changes in the cutoff wavelength cannot reflect the position of maximum reflectance. However, if we use EMT [33], we will be able to obtain a spectrum of reflection versus wavelength much more quickly than using full-wave simulations. Figure 3c and Figure 5d–f show the calculated transmission versus wavelength from EMT. The peak position for reflectance changes for different fill fraction and corresponds well to the positions of the reflection window for Figure 5a–c. However, both the magnitude and the width of reflectance predicted by EMT do not agree well with full-wave simulations. Thus, EMT also has its limits when it comes to predicting exact optical properties of LCGs. In this work, we did not explore the possibility of LCGs to have broadband reflectance but it is certainly a good topic to look into in a future study.

## 5. Conclusions

In conclusion, we have demonstrated theoretically and experimentally that we can design angular and wavelength-selective filters with LCGs. LCGs possess properties of dual mode interference and follow the effective-medium theory just like HCGs. However, LCGs do not have complicated mode dispersions or distributions unlike HCGs, making it easier for LCGs to be realize a single reflective window with larger dimensional tolerance. Our initial experiments have demonstrated that LCGs have good optical performance and will be potentially suitable for photon management in lighting and solar cells.
